# HER2/positive and HER2/low in inflammatory breast cancer recurrence

**DOI:** 10.25122/jml-2022-0213

**Published:** 2022-12

**Authors:** Oleksii Volodimirovich Movchan, Irina Yuriivna Bagmut, Andriy Fedorovich Shipko, Ivan Ivanovich Smolanka (Senior), Michael Ivanovich Sheremet, Igor Leonidovich Kolisnyk, Oleksandr Vasyliovych Bagmut, Andriy Oleksandrovich Lyashenko, Anton Dmitrovich Loboda, Oksana Mykolaivna Ivankova, Irina Viktorivna Dosenko, Oleksandr Volodimirovich Lazaruk, Yan Viktorovich Gyrla, Oleksandr Vyacheslavovich Bilookyi

**Affiliations:** 1 National Cancer Institute, Ministry of Health, Kyiv, Ukraine; 2 Kharkiv Medical Academy of Postgraduate Education, Kharkiv, Ukraine; 3 Department of Surgery No.1, Bukovinian State Medical University, Chernivtsi, Ukraine; 4 Faculty of Computer Sciences, Karazin Kharkiv National University, Kharkiv, Ukraine; 5 Department of Pathology, Bukovinian State Medical University, Chernivtsi, Ukraine

**Keywords:** HER2-low, inflammatory breast cancer, therapy marker, relapse

## Abstract

This study aimed to investigate the impact of HER2-low on the risk of recurrence in individuals with inflammatory breast cancer (IBC). 60 females with HER2-low and HER2-positive IBC underwent surgery between July 2020 and July 2022. Patients were divided into three groups of 20 patients: (1) HRplus/HER2-, (2) HRplus/HERplus, and (3) HR-/HER2plus. All patients underwent chemotherapy in adjuvant mode, following this scheme: TCH=docetaxel and carboplatin plus Herceptin (HER2 target – 4 mg/kg as the loading dose and 6 mg/kg as subsequent doses throughout every 21 days, entire 52 weeks of Herceptin therapy). HRplus/HERplus group had an OS of 76.9% compared with 77.0% in the group with the HRplus/HER2plus subtype and 74.4% in the HR-/HER2plus group. Moreover, recurrence-free survival was 19.1% for the HRplus/HER2- group, 21.3% for the HRplus/HERplus group, and 11.7% for the HR-/HER2plus group. In our study, patients with HER2-low IBC could acquire a perfect response with preliminary systemic therapy, without disease progression or with stable disease on target alone. Further examination is important to decide on the most effective treatment regimens, in addition to mixing chemotherapy with HER2-low-focused on agents.

## INTRODUCTION

Human epidermal growth factor receptor 2 (HER2)-positive breast cancer cells are more prevalent in inflammatory breast cancer (IBC), roughly 35% *vs*. 20% in other forms [1, 2]. Some can assert that IBC has distinct clinicopathological and molecular characteristics [3]. Compared to other forms, IBC identified in women at a younger age has more regrettable and aggressive results, with 2 times the survival time in the third stage as opposed to the fourth stage [4]. In addition, IBC patients have a higher relapse frequency than other forms (approximately 20% *vs*. 4%) [5]. Favorable results in HER2/positive ascribed excellent therapeutic results for targeting HER-2 agents [6].

As per the 2018 ASCO (American Society of Clinical Oncology) guideline update, breast cancers are HER2/positive if there is confirmation of HER2/overexpression by immunohistochemistry (IHC) test (score - 3+). 2+ IHC needs characterization status, with an extra survey for specific evaluation. IHC 0 and 1+, or IHC 2plus breast cancer, are HER2/negative; thus, no targeted treatment is recommended. In-situ hybridization (ISH) is a laboratory technique to identify specific genes or proteins within tissue samples. By measuring the extent of the HER2 gene and the number of copies of the HER2 protein on the cancer cells, it may be possible to determine whether the cancer is HER2-positive. If the HER2/CEP17 extent is <2.0 and the HER-2 copy range from 4.0 to 5.9 on cell, HER2/positive breast cancer can be confirmed on ISH [7].

A concept that uses a combination known as IHC 1plus or 2plus and known as negative ISH was proposed as HER2-low IBC. However, no medicines disrupting the HER2 pathway have been found to have a therapeutic benefit for HER2-low breast cancers [8].

The IHC/ISH approach involves using both IHC and ISH to determine the status of the tumor as HER2-positive or HER2-negative. Immunohistochemical results can sometimes be controversial, and the IHC/ISH approach can help to provide a more definitive answer about the HER2 status of a tumor [9].

HER2/low breast cancers are usually hormone receptor positive (HR+) and make up the majority of breast cancers (65–83%), while the rest are HR/negative and show a predominance of triple-negative breast cancers (TN-BC) [10, 11].

Endocrine treatment is not effective in treating HER2/negative IBC, and treatment for HR/plus is also known to be limited [12, 13] due to the dependence on standard chemotherapy. Consequently, survival rates are generally lower for IBC than for other types of breast cancer [14, 15].

This article examined the evolution of HER2 treatment in HER2-low IBC, suggesting a way to characterize the influence of HER2-low IBCs on relapses. It is important to accurately determine the level of HER2 expression in breast cancer to choose the most appropriate treatment approach. Traditionally, breast cancer has been classified as either HER2-positive or HER2-negative based on the presence or absence of the HER2 protein. However, recent research has identified a subgroup of breast cancers with intermediate levels of HER2 expression, known as HER2-low. These tumors may be difficult to classify as either HER2-positive or HER2-negative using traditional methods, which may impact the treatment choice.

This study aimed to investigate the impact of HER2-low on the risk of recurrence in individuals with inflammatory breast cancer (IBC).

## MATERIAL AND METHODS

The study was carried out in accordance with the Helsinki Declaration, GCP (Good Clinical Practice), and Ukrainian legislation on medications. We included 60 females with HER2/low and HER2/positive inflammatory breast cancer treated in our institute between July 2020 and July 2022. Patients were divided into three groups of 20 patients: (1) HRplus/HER-2-, (2) HRplus/HER-2plus, and (3) HR-/HER-2plus. All patients underwent chemotherapy in adjuvant mode, in accordance with the following scheme: TCH=docetaxel and carboplatin plus Herceptin (HER-2 target – four mg/kg as the loading dose and 6mg/kg as subsequent doses throughout every 21 days, entire 52 weeks of Herceptin therapy) [16, 17].

Patients were under observation for 24 months. Medical records and pathology reports were examined for pre-determinate parameters: tumor size (T), nodal status (N), histologic grade (G), HER2 expression (0; 1plus; 2minus; 2plus; 3plus), estrogen receptor (ER), and progesterone receptor (PR), index Ki-67.

HER2-low tumors were described using IHC as plus1 or IHC plus2/ISH -. HER2-zero was recorded when it was IHC-0. ER and PR staining were introduced with a score ranging from 0 to 3, according to the modified version of the H-score method. The intensity of hormone receptors was reported as percentages [18]. The intensity of hormone receptor staining was classified into 3 categories: weak (0<ER/PR≤1), intermediate (1<ER/PR≤2), and strong (ER/PR>2). The histologic grade was determined using the Nottingham Histologic Scoring system [19].

The condition of the patients corresponded to generally accepted norms of the Ministry of Health for patients with this pathology. Overall survival (OS) was described as the interval between the date of diagnosis and death from any motive. Recurrence-free survival (RFS) was defined as the time from surgery to disease relapse. HER-2 immunohistochemistry was performed according to the generally accepted methodology (Table 1). Each of these outcomes necessitates specific patient management [20].

**Table 1:** HER2 Testing by Validated IHC Assay.

Status	Score	Significance	Reflex HER2 FISH
**Positive**	3+	Uniform intense membrane staining of >30% of invasive tumor cells	No
**Equivocal**	2+	Complete membrane staining, nonuniform or weak in intensity in at least 10% of the cells or intense complete membrane staining in 30% or less of tumor cells	Yes
**Negative**	1+	Weak or incomplete membrane staining in any proportion of tumor cells	No
**Negative**	0	No staining	No

IHC – indicates immunohistochemistry; FISH – fluorescence in situ hybridization [Dako. HercepTest™, Code K5204. Instruction for Use, (PD04086US_02/K520421-5)].

Fluorescence microscopy was used to perform fluorescence staining (FISH) on materials from paraffin blocks [21]. An HER-2 FISH positive result is defined as an average of more than 6 HER-2 gene copies for test systems without an interior control probe or HER-2/CEP 17 ratio of greater than 2.2, where CEP17 is a centromeric probe for chromosome 17 on which the HER-2 gene is located. For HER-2 FISH assays, the equivocal range is defined as HER/CEP ratios from 1.8 to 2.2 or average gene copy number ranging from 4.0 to 6.0 for all systems except an internal control. Negative HER-2 FISH amplification is defined as an HER-2/CEP17 ratio of less than 1.8 or fewer than 4 copies of the HER-2 gene per nucleus in systems lacking an interior control probe. Tumor samples containing insufficient invasive cancer, tissue not fixed in buffered formalin or fixed for more than 48 hours, and background autofluorescence make a specimen unreadable [22].

In situ hybridization is proposed as the preferred method for HER2 gene testing because of its advantages over IHC [23, 24]. We used different samples to determine the number of current copies of a specific region of DNA for distinct locus rearranged as part of chromosomal changing [25].

### Statistical analysis

We used Mann–Whitney U-test to compare two or more groups. Kaplan-Meier curves were used to estimate survival data, while the p-value associated with the long-rank test was calculated. Fisher's exact test was used to evaluate associations between categorical data, and Spearman correlation was used to determine the correlation between hormone status and HER2 gene expression levels. All analyses were conducted in R (version 4.0.2).

## RESULTS

All 20 patients in the HRplus/HER-2- group, 20 in the HRplus/HERplus group, and 20 in the HR-/HER-2plus group were included in the analysis without missing data. The variables are summarized in Table 2. There were 55.8% postmenopausal patients in the HRplus/HERplus group and 62.1% in the HRplus/HER2plus and HR-/HER2- groups. Furthermore, patients in the HR-/HER-2- group performed significantly worse than those in the HRplus/HER2plus group (p<0.05). Relapses revealed that the incidence in HR-positive groups reached up to 23.4%, considerably higher than in HR-negative groups.

**Table 2:** Variables of patients in subgroups, follow-up 24 months.

Variables	HR-/HER2- N=20 (%)	HR+/HER2+ N=20 (%)	HR+/HER+ N=20 (%)	P-value*
**Patients' age, years**				
<35	1 (4.1)	2 (12.9)	1 (4.1)	0.179
35–49	4 (20.4)	7 (33.3)	4 (20.4)	
50–64	8 (42.1)	7 (33.3)	2 (12.9)	
≥65	7 (33.3)	4 (20.5)	13 (62.1)	
**Grade**				
Well	1 (4.1)	0 (0)	0	0.003
Moderately	7 (33.3)	5 (25)	2 (12.9)	
Poorly	8 (41.2)	13 (62.1)	13 (62.1)	
Undifferentiated	4 (20.4)	2 (12.9)	5 (25)	
**Stage**				
II	5 (25)	4 (20.4)	4 (20.4)	0.742
III	15 (75)	16 (79.6)	16 (79.6)	
**Tumor size (millimeter)**				
≤10	1 (4.1)	2 (12.9)	4 (20.4)	0.884
10–20	8 (42.2)	1 (4.1)	2 (12.9)	
20–50	5 (25)	4 (20.4)	13 (62.1)	
>50	6 (28.7)	13 (62.1)	1 (4.1)	
**Surgery**				
Mastectomy	7 (33.3)	4 (20.4)	5 (25)	0.690
BCS (breast-conserving surgery)	13 (66.7)	16 (79.6)	15 (75)	
**Result of the treatment**				
Without recurrence	16 (79.6)	18 (95.9)	17 (85.2)	<0.001
With recurrence	4 (20.4)	1 (4.1)	3 (14.8)	

* – p-values calculated by Pearson Chi squared testing; Bold if statistically significant, p<0.05; HR – hormone receptor.

### Survival analysis

The overall survival rate (OS) was 76.9% in the HRplus/HERplus group compared to 77.0% in the HRplus/HER-2plus and 74.4% in the HR-/HER2plus group. Moreover, RFS was 19.1% for the HRplus/HER-2- group, 21.3% for the HRplus/HERplus group, and 11.7% for the HR-/HER2plus group.

**Figure 1:**
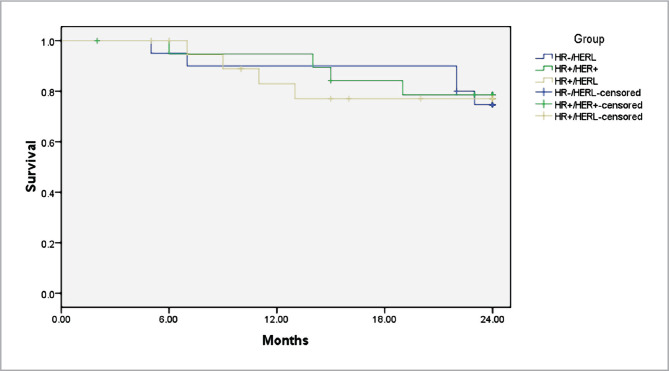
Weighted Kaplan–Meier curves of overall survival (OS) after treatment of inflammatory breast cancer based on the HER2 status.

**Figure 2:**
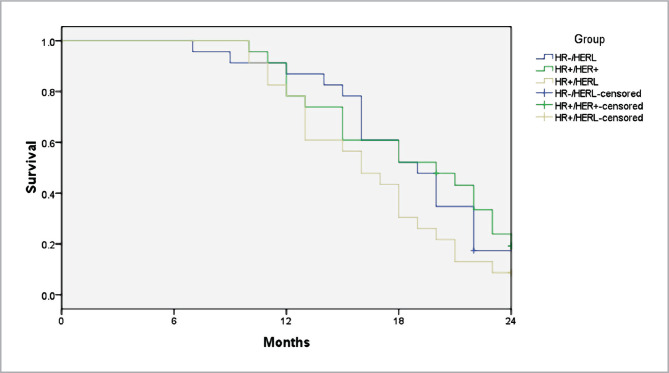
Kaplan–Meier curves of recurrence-free survival (RFS) after treatment of inflammatory breast cancer based on the molecular subtypes.

A multivariate analysis using Cox regression was performed based on the Kaplan–Meier results. All prognostic factors predicted OS and RFS on multivariate analysis (Figure 1 and Figure 2). HER-2 positivity was associated with better survival (OS, adjusted hazard ratios (aHR)=0.334; RFS, aHR=0.327, P<0.05) (Figure 1 and Figure 2). We found the same results for the estrogen receptor (ER).

## DISCUSSION

HER2 targeted treatment is effective not only in HER2/positive patients but also in HER2/low patients [26], as evidenced by our favorable results. HER-2 positivity was associated with better survival (OS, adjusted Hazard Ratios (aHR)=0.334; RFS, aHR=0.327, p<0.05). The ORR was 37% (95% CI, 24.3–51.3%), with a median duration of response of 10.4 months, in a refractory HER-2/positive IBC patients' cohort and a heavily pretreated cohort of HER2-low IBC patients, which corresponds to our study results.

According to Yu K. et al. [27], patients with tumors that were ER-positive had a higher likelihood of survival compared to those with ER-negative tumors, corresponding to our study. The findings revealed that patients with HR-positive IBC had a significantly higher survival rate (median OS 31 months) than those with HR-negative IBC (24 months). Thus, research has shown that HER2/low IBC is a heterogeneous disease that can exhibit different molecular characteristics and behaviors depending on the specific subtype associated with distinct prognostic outcomes compared to other breast cancers and systemic therapies such as hormonotherapy and anti-HER-2 targeted therapy were effective in treating IBC.

IBC surgical treatment has been lately debated. Patients with IBC traditionally had mastectomies. Some patients reached complete pathological response (pCR) with neoadjuvant chemotherapy or radiation therapy (RT) and started breast-conserving surgery (IBCS). According to one study, IBC patients who responded well to systemic therapy may benefit from IBCS [28]. As a result, more extensive observations and studies should be carried out to confirm these findings. This study presented the molecular characteristics of primary HER2/low IBC and a comprehensive analysis of its prognosis. HER2/low IBC was diagnosed in 57% of patients from our cohort, with the majority (77%) HRplus, corresponding with previous reports [29].

Our findings show that HER-2/low IBC tumors, as opposed to HER-2-zero and Her-2/positive tumors, can be identified as a new subset of inflammatory breast cancer using standardized IHC. HER-2/low tumors have a distinct biology that influences treatment response and prognosis. This is especially important for hormone-receptor-negative tumors that are resistant to treatment. Our findings lay the groundwork for a better understanding of the biology of breast cancer subtypes as well as the development of future diagnostic and therapeutic strategies [30].

## CONCLUSIONS

Over thirty years after the discovery of the HER2 protein, anti-HER2 therapy regimens are only now going to be implemented in the HER2/low IBC population.

Pointers currently advocate a two-sided model (HER-2-beneficial *vs*. harmful) to inform the treatment approach. However, a significant percentage of patients (over 50%) classified as HER2-negative are actually Her2/low, a population with a high unmet clinical need. Despite the limitations of older drugs, a new technology of anti-HER-2 retailers has recently demonstrated favorable symptoms and scientific activity for protection in HER2/low.

Based on our study, patients with HER2/low IBC could acquire a perfect response with preliminary systemic therapy, without disease progression or with stable disease on target alone. Further studies should focus on the highest quality series of the more modern regimens, in addition to the mixing of chemotherapy with HER-2/low-focused on agents.

## Personal thanks

We want to thank professor Irina Yurievna Bagmut.

## References

[1] van Uden D, van Maaren M, Strobbe L, Bult P (2019). Metastatic behavior and overall survival according to breast cancer subtypes in stage IV inflammatory breast cancer. Breast Cancer Res.

[2] Liu J, Chen K, Jiang W, Mao K, Li S (2017). Chemotherapy response and survival of inflammatory breast cancer by hormone-receptor-and HER-2-defined molecular subtypes approximation: an analysis from the National Cancer Database. J Cancer Res Clin Oncol.

[3] Faldoni F, Rainho C, Rogatto S (2020). Epigenetics in Inflammatory Breast Cancer: Biological Features and Therapeutic Perspectives. Cells.

[4] Lim B, Woodward W, Wang X, Reuben J, Ueno N (2018). Inflammatory breast cancer biology: the tumour microenvironment is key. Nat Rev Cancer.

[5] Dano D, Lardy-Cleaud A, Monneur A, Quenel-Tueux N (2021). Metastatic inflammatory breast cancer: survival outcomes and prognostic factors in the national, multicentric, and real-life French cohort (ESME). ESMO Open.

[6] Ge J, Overmoyer B (2021). Prolonged Survival in Patients with Metastatic Her-2/positive Inflammatory Breast Cancer: A Case Report and Review of the Literature. Case Rep Oncol.

[7] Tarantino P, Hamilton E, Tolaney S, Cortes J (2020). Her-2/low Breast Cancer: Pathological and Clinical Landscape. Journal of Clinical Oncology.

[8] Agostinetto E, Rediti M, Fimereli D, Debien V (2021). HER2-Low Breast Cancer: Molecular Characteristics and Prognosis. Cancers (Basel).

[9] Thibodeau S, Voutsadakis I (2019). The Oncotype Dx Assay in ER-Positive, HER-2-Negative Breast Cancer Patients: A Real-Life Experience from a Single Cancer Center. Eur J Breast Health.

[10] Schettini F, Chic N, Brasó-Maristany F, Paré L (2021). Clinical, pathological, and PAM50 gene expression features of Her-2/low breast cancer. NPJ Breast Cancer.

[11] Fehrenbacher L, Cecchini R, Geyer C, Rastogi P (2020). NSABP B-47/NRG Oncology Phase III Randomized Trial Comparing Adjuvant Chemotherapy With or Without HER-2 target in High-Risk Invasive Breast Cancer Negative for HER-2 by FISH and With IHC 1plus or 2. J Clin Oncol.

[12] Cardoso F, Senkus E, Costa A, Papadopoulos E (2018). 4^th^ ESO-ESMO International Consensus Guidelines for Advanced Breast Cancer (AIBC 4). Ann Oncol.

[13] Kaufman B, Trudeau M, Awada A, Blackwell K, Bachelot T (2009). Lapatinib monotherapy in patients with HER-2-overexpressing relapsed or refractory inflammatory breast cancer: final results and survival of the expanded HER-2plus cohort in EGF103009, a phase II study. Lancet Oncol.

[14] Waks A, Winer E (2019). Breast Cancer Treatment: A Review. JAMA.

[15] Caswell-Jin J, Plevritis S, Tian L, Cadham C (2018). Change in Survival in Metastatic Breast Cancer with Treatment Advances: Meta-Analysis and Systematic Review. JNCI Cancer Spectr.

[16] Referenced with permission from the NCCN Clinical Practice Guidelines in Oncology (NCCN Guidelines®) for Breast Cancer V.1.2019. © National Comprehensive Cancer Network, Inc. 2019. All rights reserved. http://www.NCCN.org.

[17] (2021). Herceptin Prescribing Information.

[18] Mutai R, Barkan T, Moore A, Sarfaty M (2021). Prognostic impact of Her-2/low expression in hormone-receptor positive early breast cancer. Breast.

[19] American Cancer Society (2019). Breast Cancer Staging. http://www.cancer.org/cancer/breastcancer/detailedguide/breast-cancer-staging.

[20] Jorgensen J, Winther H, Askaa J, Andresen L, Olsen D (2021). Companion Diagnostic with Significant Clinical Impact in treatment of Breast and Gastric Cancer. Front Oncol.

[21] Chrzanowska N, Kowalewski J, Lewandowska M (2020). Use of Fluorescence In Situ Hybridization (FISH) in Diagnosis and Tailored Therapies in Solid Tumors. Molecules.

[22] Radziuviene G, Rasmusson A, Augulis R, Lesciute-Krilaviciene D (2017). Automated Image Analysis of HER-2 Fluorescence In Situ Hybridization to Refine Definitions of Genetic Heterogeneity in Breast Cancer Tissue. Biomed Res Int.

[23] Atabati H, Raoofi A, Amini A, Farahani R (2018). Evaluating HER-2 Gene Amplification Using Chromogenic In Situ Hybridization (CISH) Method In Comparison To Immunehistochemistry Method in Breast Carcinoma. Open Access Maced J Med Sci.

[24] Bagmut I, Movchan O, Sheremet M, Smolanka I (2022). Characteristics of certain genetical and biological properties of carcinogenesis in the development of inflammatory breast cancer with type 2 diabetes mellitus and tumor relapse. Rom J Diabetes Nutr Metab Dis.

[25] Shakoori AR (2017). Fluorescence In Situ Hybridization (FISH) and Its Applications. Chromosome Structure and Aberrations.

[26] Eiger D, Agostinetto E, Saúde-Conde R, de Azambuja E (2021). The Exciting New Field of Her-2/low Breast Cancer Treatment. Cancers (Basel).

[27] Viale G, Marra A, Curigliano G, Criscitiello C (2019). Toward precision medicine in inflammatory breast cancer. Translational cancer research.

[28] Yu K, Cai Y, Wu S, Shui R, Shao Z (2021). Estrogen receptor-low breast cancer: Biology chaos and treatment paradox. Cancer Commun (Lond).

[29] Smolanka I, Bagmut I, Sheremet M, Lyashenko A (2021). Delayed breast reconstruction with tram-flap and various modifications after radical mastectomy. J Med Life.

[30] Denkert C, Seither F, Schneeweiss A, Link T (2021). Clinical and molecular characteristics of Her-2/low-positive breast cancer: pooled analysis of individual patient data from four prospective, neoadjuvant clinical trials. Lancet Oncol.

